# *Apolipoprotein E* promoter genotypes are not associated with white matter hyperintensity development in high-altitude careers

**DOI:** 10.1186/s13104-019-4654-9

**Published:** 2019-09-24

**Authors:** Richard R. Chapleau, CharLee A. Martin, Summer R. Hughes, James C. Baldwin, John Sladky, Paul M. Sherman, Michael Grinkemeyer

**Affiliations:** 1 Aeromedical Research Department, Applied Technology and Genomics Division, Wright-Patterson AFB, U.S. Air Force School of Aerospace Medicine, Dayton, OH USA; 2 Aeromedical Research Department, Operational Health and Performance Research Division, Wright-Patterson AFB, U.S. Air Force School of Aerospace Medicine, Dayton, OH USA; 3 59th Medical Wing, Department of Neurology, Joint Base San Antonio-Lackland, San Antonio, TX USA; 4 59th Medical Wing, Department of Neuroradiology, Joint Base San Antonio-Lackland, San Antonio, TX USA

**Keywords:** Apolipoprotein E, High altitude acclimatization, Neuroprotection, Genotype screening

## Abstract

**Objective:**

This study sought to determine if there is an association between variants in the *apolipoprotein E* (*ApoE*) promoter regions and development of white matter hyperintensities (WMH) in military subjects who have been exposed to high altitude. In an earlier study, we found that *ApoE* status did not correlate with WMH development, and here we hypothesized that regulation of APOE protein expression may be protective.

**Results:**

Our cohort of 92 subjects encountered altitude exposures above 25,000 feet mean sea level through their occupations as pilots or altitude chamber technicians. Using Taqman-style polymerase chain reaction genotyping and t-tests and two-way analyses of variance we found no significant association between *ApoE* promoter genotypes and the presence, volume, or quantity of WMHs after high altitude exposure. Taken together, the observations that neither *ApoE* genotype status nor promoter status are associated with WMH properties, we believe that the mechanism of action for developing WMH does not derive from *ApoE,* nor would therapies for *ApoE*-mediated neurodegeneration likely benefit high altitude operators.

## Introduction

White matter hyperintensities (WMH) are small lesions in the brain that appear as bright signals on an MRI (magnetic resonance imaging) radiograms [1]. These lesions have been associated with decreased cognitive performance [2] and decreased cerebral blood flow [3]. For those rare populations that encounter extreme high altitudes (greater than 25,000 feet above mean sea level), there is a real risk of repetitive non-hypoxic hypobaria resulting in neurological defects. A self-report survey of high altitude aviators found an apparent prevalence of 75% for decompression sickness [4]. As manned space flight, high-altitude military missions, and high-altitude recreational activities increase, there is a need to better understand how these lesions appear and how to identify individual susceptibility to WMH development.

Previous studies from our group investigated the downstream effects of WMH [5, 6] as well as possible genetic predispositions to developing WMH after altitude [7]. A number of studies have shown that genetic variants of the *apolipoprotein E (ApoE)* gene have been associated with neurodegenerative disorders [8–10]. *ApoE2* status correlates with increasing brain WMHs [11], and *ApoE* genotype status is also associated with traumatic brain injury recovery [10] as well as risk of hemorrhagic and ischemic cerebrovascular disease [11]. *ApoE4* is associated with an increased risk of Alzheimer’s disease [12, 13], and yet is protective in people who consume high levels of fish [14]. In a previous study, we did not find a significant association between *ApoE* genotype status and altitude-associated WMH [7].

Considering the evidence for genetic associations of Alzheimer’s disease and WMH burden, and relationships between *ApoE* and Alzheimer’s disease, we elected to extend our earlier, null result, candidate gene study to include promoter regions of the *ApoE* gene. A recent multi-ethnic genome wide association study identified eight single nucleotide polymorphisms (SNPs) in five unique loci as genome-wide significant with association to WMH burden, including both novel SNPs and novel loci [15]. As a pilot study, here, we targeted two of those variants with strong evidence of association between neurodegenerative disease and neurological impact. The − 219 T/C variant (rs405509) was associated with increased risk of late onset Alzheimer’s disease in combination with *ApoE4* status [16]. A second study was able to confirm the association of this SNP as well as to confirm the association of the − 427 T/G variant (rs769446) [17]. More recently, a meta-analysis of 23 Alzheimer’s disease genetics studies confirmed the association of rs769446 but called into question the validity of rs405509 [18]. Our hypothesis was that these promoter variant**s** would be associated with the development of WMH after exposure to altitudes above 25,000 feet.

## Main text

### Methods

The phenotyping and genotyping methods used in this study replicate our approach in the earlier *ApoE* status study [7]. The methods are summarized here for clarity.

#### Study design and MRI data collection

Subjects were recruited from a research study investigating WMH burden in high altitude aircrew [5, 6]. From the initial MRI study, we were able to enroll 44 pilots and 48 altitude chamber operators. All participants were healthy, adult males with MRIs collected in the original study. MRI radiographs were measured from WMH burden and data were combined with genotype data in fully de-identified manner.

#### Sample collection and laboratory analysis

Purple-top blood tubes were sent directly to participants who were instructed to report to a military medical treatment facility for phlebotomy. The facility then sent blood to our lab for DNA extraction using the Promega Maxwell 16 Blood DNA Purification kit and genotyping by Thermo Fisher polymerase chain reaction genotyping assays (rs405509 and rs769446). The genotyping was performed on a Thermo Fisher/ABI 7500 FAST thermocycler using the instrument’s standard PCR genotyping parameters.

#### Statistical analysis

Statistics were performed in GraphPad Prism 7.0c. The factors for two-way ANOVAs were genotype (primary) and each of the phenotypes independently as second factors. Where applicable, multiple t-tests were performed assuming similar scatter and using a 10% false discovery rate according to the two-stage step-up method of Benjamini, Krieger, and Yekutieli [19]. Additionally, Fisher’s exact test was used to determine if the observed allele and genotype distributions were different from global populations and also to determine odds ratios, as described in the results section.

### Results

Sample size calculations resulted in groups as small as 8 per genotype required using the average WMH lesion volume for rs769446 groups (0.05 ± 0.06 heterozygotes vs. 0.17 ± 0.47 T-homozygotes). We observed 19 heterozygotes and 73 T homozygotes in these groups. Performing sample size calculations for either lesion count or lesion volume using rs405509 homozygotes (T/T vs. G/G) or alleles (T vs. G) resulted in very large cohort sizes (1202 subjects per homozygous group when using mean lesion count as the classifier). The average WMH counts for the T/T, T/G, and G/G genotype groups at rs405509 were 7.6 ± 10.5 (n = 16), 8.3 ± 16.6 (n = 52), and 6.4 ± 12.9 (n = 24), respectively. Therefore, this study was underpowered to identify a statistically significant difference at this locus. As mentioned in the previous work, the population in this study includes a representative sampling of between 50 and 90% of currently qualified high altitude pilots [13], and approximately 1% of the total population of high altitude pilots ever to have flown. Therefore, our results from both studies are expected to reflect the true population.

Compared to other populations, we observed significantly more heterozygotes at rs405509 than in either the global or American populations (P = 0.017) [12]. We found 55.4% heterozygotes in our study compared to 45.2% globally and 46.4% in US populations. This appears to derive from a decrease in the G homozygotes where we found 16.3% in our study vs. 30.2% and 26.5% in the other populations, respectively. Our population contained 28.3% T homozygotes, consistent with the 24.6% and 27.1% values for the respective populations. Overall, the allele frequencies were not significantly different when assessed using Fisher’s exact test (P = 0.41). While there were no C homozygotes in our population for rs769446, we did observe 20.7% heterozygotes and 79.3% T homozygotes, which is consistent with a C homozygote frequency of 0.4% globally and of 0.0% in the US [12].

In a two-way analysis of variance between genotype and WMH lesions, neither the interaction nor the genotype effects are considered significant, accounting for no more than 3.1% of the total variance. The phenotypes were the greatest sources of variance, accounting for 30.8% and 36.4% for rs405509 and rs769446, respectively. Multiple t-tests to evaluate possible associations between genotype and WHM lesion count or volume revealed no significant associations (Table 1).

**Table 1:** T-test comparisons

Genotype comparison (rs405509)	WMH count *p* value	WMH volume p-value	Genotype comparison (rs769446)	WMH count p-value	WMH volume p-value
T/T vs. G/G	0.99	> 0.99	C/C vs. T/T	N/A^*^	N/A^*^
T/T vs. T/G	0.97	> 0.99	C/C vs. C/T	N/A^*^	N/A^*^
T/G vs. G/G	0.95	> 0.99	C/T vs. T/T	0.19	0.28

^*^There were no C/C genotypes in the study

**Fig. 1:**
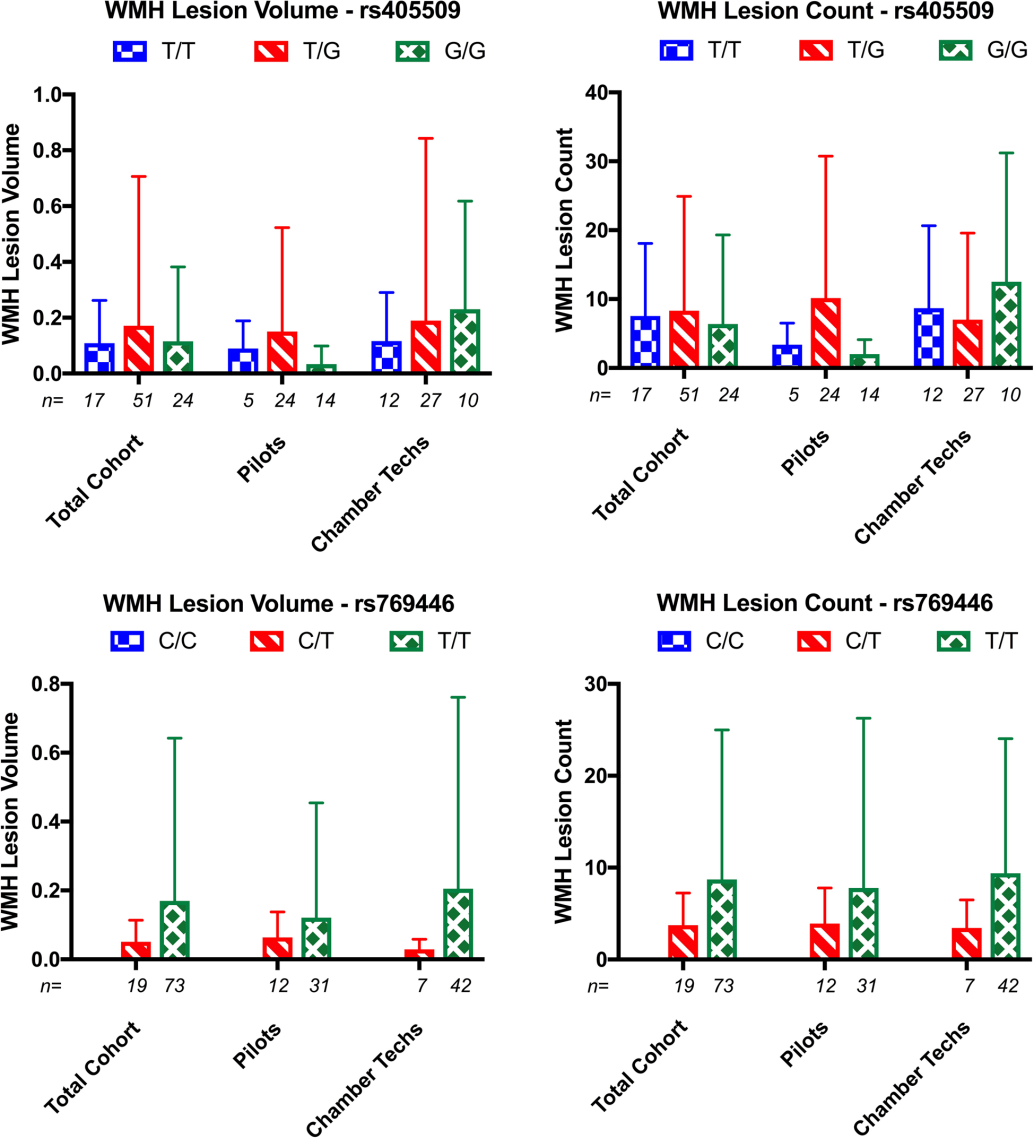
White matter hyperintensity (WMH) metrics by exposure group and *ApoE* promoter genotypes. There are no significant differences by any of the three observed genotypes at rs405509 (top) for individual groups or the cohort as a whole (total cohort). Neither are there any significant associations observed at rs769446 (bottom). Error bars represent standard deviation from the mean; sample sizes are indicated as italicized numbers beneath the respective bar

When broken out by career field, the lack of an association between *ApoE* promoter genotype status and WMH variables becomes visually apparent (Fig. 1). As previously mentioned, we failed to observe statistical significance between the differences of the means by multiple t-tests at either locus for WMH lesion volume or count. There appears to be a trend, although not significant, at rs769446 that the C carriers have smaller volume and lower count. Although this observation comes with the caveat that there were no C homozygotes in the study and only 19 heterozygotes.

**Fig. 2:**
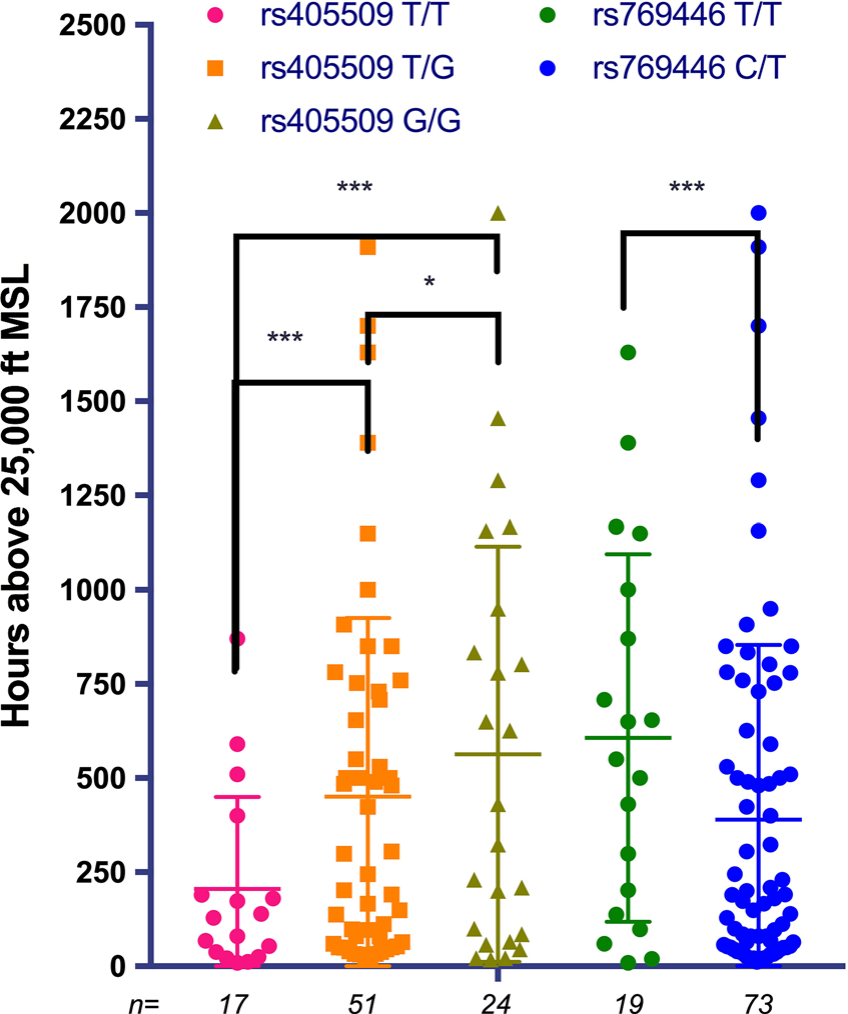
Average hours accumulated above 25,000 feet MSL by genotype. The average values from left to right are 205, 451, 563, 607, and 390 h, respectively. Error bars represent standard deviation from the mean; sample sizes are indicated as italicized numbers beneath the respective bar; significance was determined by Fisher’s exact test (* P < 0.05, *** P < 0.001)

Similarly to the previously published results regarding *ApoE* genotype status, we found a positive association between promoter genotypes and hours flown above 25,000 feet mean sea level (Fig. 2). Having a G allele at rs405509 is associated with an odds ratio of 1.6 for accumulating greater than 450 h above 25,000 feet (the mean value for the heterozygotes). As alluded to above, there were only two genotypes for rs769446 in the study and only 19 heterozygotic subjects in the study, an odds ratio calculation was not possible.

### Discussion

As more high performance aircraft are being developed with service ceilings greater than 50,000 feet, and as commercial space flight is a reality, there is a clear need to understand the impacts of flight on physiology and to identify how an individual may be more resilient or susceptible to these impacts. While we investigated altitude exposure above 25,000 feet on WMH development, repetitive exposure to altitudes above 10,000 feet is commonly seen in recreational parachuting and commercial air travel is pressurized to 8000 feet. Therefore, gaining a better understanding of the genetic risk or resilience markers associated with high altitude exposure could have a wide ranging medical impact.

The broad potential for individual exposures to elevated altitudes, defined here as those altitudes at which a person is not normally acclimated, to negatively impact health is precisely why we feel these studies provide important information. The earlier study determined that *ApoE* genotype status was not associated with WMH metrics, though it did identify that *ApoE2* carriers were found to have accumulated a higher number of hours above 25,000 feet [7]. This is consistent with our observation here that G carriers in the promoter region at rs405509 also accumulated greater high altitude exposures. Whether these observations are indicative of a protective effect at altitude remains untested, but it could provide insight to future studies at lower altitudes.

As a promoter variant, the role of rs405509 in human health is complex. The G allele of this variant has been associated with increased ApoE protein levels [20]. Furthermore, G carriers also have been shown to have higher cognitive functions in attention and executive function [21] and lower risk of incurring multiple concussions [22]. Taken together, these observations suggest that increased levels of ApoE protein could be protective against neurological deficit caused by chronic high altitude exposure. While we have now investigated the possible association of *ApoE* genotype and two promoter variants with WMH development, we have not directly interrogated protein levels. In living subjects the ApoE protein levels can be checked in either circulating blood or cerebrospinal fluid [23]. We collected whole blood in our study but do not have the capabilities to quantify protein levels, therefore we extend our interpretation using the discussions and observations of other researchers.

The cognitive decline seen in individuals with increased WMH burden and the cognitive decline found in persons with the *ApoE* promoter genotypes tested here do not seem to be associated. From our two studies, it appears that *ApoE* is not a contributing risk factor for altitude-induced neurological damage. The study by Verhaaren and co-workers [15] suggests several more polymorphisms associated with white matter hyperintensities which may provide additional testable hypothesis to identify genetic risk markers for altitude-induced WMH burden. Here we studied two promoter variants within the same linkage disequilibrium (LD) block, in light of our negative association results and other studies finding associations in nearby LD blocks, it may be possible that evaluating the − 491 variant (rs449647) [24]. Furthermore, their work shows the power of genome wide association studies for identifying risk markers, and could be extended to identify positive markers of resilience against the effects of altitude on WMH load. Performing such studies with individuals exposed to a range of altitudes could provide critical information for healthcare providers in aerospace and mountain medicine.

## Limitations

The primary limitations of this study are: (1) the small sample size (n = 92), and (2) the emphasis on a single gene and its promoter. While increasing the number of participants is not practical as the career fields are not growing rapidly, the continuous nature of the phenotype variables makes it possible to perform genome-wide analyses for a hypothesis generating study on this cohort size if the heritability of the WMH trait is high enough (approximately 20–30%).

## Data Availability

Summary data from this study, including genotype and hyperintensity measurements, are available from the corresponding author upon reasonable request.

## Abbreviations

*ApoE*: *apolipoprotein E*
MRI: magnetic resonance imaging
WMH: white matter hyperintensity

## References

[1] Hase Y, Horsburgh K, Ihara M, Kalaria RN (2018). White matter degeneration in vascular and other ageing-related dementias. J Neurochem.

[2] Habes M, Erus G, Toledo JB, Zhang T, Bryan N, Launer LJ, Rosseel Y, Janowitz D, Doshi J, Van der Auwera S, von Sarnowski B, Hegenscheid K, Hosten N, Homuth G, Völzke H, Schminke U, Hoffmann W, Grabe HJ, Davatzikos C (2016). White matter hyperintensities and imaging patterns of brain ageing in the general population. Brain.

[3] Bahrani AA, Powell DK, Yu G, Johnson ES, Jicha GA, Smith CD (2017). White matter hyperintensity associations with cerebral blood flow in elderly subjects stratified by cerebrovascular risk. J Stroke Cerebrovasc Dis.

[4] Bendrick GA, Ainscough MJ, Pilmanis AA, Bisson RU (1996). Prevalence of decompression sickness among U-2 pilots. Aviat Space Environ Med.

[5] McGuire SA, Sherman PM, Brown AC, Robinson AY, Tate DF, Fox PT, Kochunov PV (2012). Hyperintense white matter lesions in 50 high-altitude pilots with neurologic decompression sickness. Aviat Space Environ Med.

[6] McGuire S, Sherman P, Profenna L, Grogan P, Sladky J, Brown A, Robinson A, Rowland L, Hong E, Patel B (2013). White matter hyperintensities on MRI in high-altitude U-2 pilots. Neurology.

[7] Chapleau RR, Martin CA, Hughes SR (2018). Evaluating apolipoprotein E genotype status and neuroprotective effects against white matter hyperintensity development in high-altitude careers. BMC Res Notes.

[8] Friedman G, Froom P, Sazbon L, Grinblatt I, Shochina M, Tsenter J, Babaey S, Yehuda B, Groswasser Z (1999). Apolipoprotein E-epsilon4 genotype predicts a poor outcome in survivors of traumatic brain injury. Neurology.

[9] Mayeux R, Ottman R, Maestre G, Ngai C, Tang MX, Ginsberg H, Chun M, Tycko B, Shelanski M (1995). Synergistic effects of traumatic head injury and apolipoprotein-epsilon 4 in patients with Alzheimer’s disease. Neurology.

[10] Sorbi S, Nacmias B, Piacentini S, Repice A, Latorraca S, Forleo P, Amaducci L (1995). ApoE as a prognostic factor for post-traumatic coma. Nat Med.

[11] Schilling S, DeStefano AL, Sachdev PS, Choi SH, Mather KA, DeCarli CD, Wen W, Høgh P, Raz N, Au R (2013). APOE genotype and MRI markers of cerebrovascular disease: systematic review and meta-analysis. Neurology.

[12] Corder EH, Saunders AM, Strittmatter WJ, Schmechel DE, Gaskell PC, Small GW, Roses AD, Haines JL, Pericak-Vance MA (1993). Gene dose of apolipoprotein E type 4 allele and the risk of Alzheimer’s disease in late onset families. Science.

[13] Spinney L (2014). Alzheimer’s disease: the forgetting gene. Nature.

[14] Morris MC, Brockman J, Schneider JA, Wang Y, Bennett DA, Tangney CC, van de Rest O (2016). Association of seafood consumption, brain mercury level, and APOE ε4 status with brain neuropathology in older adults. JAMA.

[15] Verhaaren BF, Debette S, Bis JC (2015). Multiethnic genome-wide association study of cerebral white matter hyperintensities on MRI. Circ Cardiovasc Genet.

[16] Lescai F, Chiamenti AM, Codemo A, Pirazzini C (2011). An APOE haplotype associated with decreased ε4 expression increases the risk of late onset Alzheimer’s disease. J Alzheimers Dis.

[17] Bizzarro A, Seripa D, Acciarri A (2009). The complex interaction between APOE promoter and AD: an Italian case-control study. Eur J Hum Genet.

[18] Xiao H, Gao Y, Liu L, Li Y (2017). Association between polymorphisms in the promoter region of the apolipoprotein E (APOE) gene and Alzheimer’s disease: a meta-analysis. EXCLI J.

[19] Benjamini Y, Krieger AM, Yekutieli D (2006). Adaptive linear step-up procedures that control the false discovery rate. Biometrika.

[20] Artiga MJ, Bullido MJ, Sastre I (1998). Allelic polymorphisms in the transcriptional regulatory region of apolipoprotein E gene. FEBS Lett.

[21] Shu N, Li X, Ma C (2015). Effects of APOE promoter polymorphism on the topological organization of brain structural connectome in nondemented elderly. Hum Brain Mapp.

[22] Tierney RT, Mansell JL, Higgins M (2010). Apolipoprotein E genotype and concussion in college athletes. Clin J Sport Med.

[23] Sofat R, Cooper JA, Kumari M (2016). Circulating Apolipoprotein E concentration and cardiovascular disease risk: meta-analysis of results from three studies. PLoS Med.

[24] Geng H, Law PPY, Ng MCY (2011). APOE genotype-function relationship: evidence of -491 A/T promoter polymorphism modifying transcription control but not Type 2 diabetes risk. PLoS ONE.

